# Modified Ideal Cardiovascular Health Status is Associated with Lower Prevalence of Stroke in Rural Northeast China

**DOI:** 10.3390/ijerph13020207

**Published:** 2016-02-06

**Authors:** Liang Guo, Xiaofan Guo, Ye Chang, Zhao Li, Shasha Yu, Hongmei Yang, Yingxian Sun

**Affiliations:** Department of Cardiology, The First Hospital of China Medical University, Shenyang 110000, China; doctorgl@126.com (L.G.); guoxiaofan1986@foxmail.com (X.G.); chang.ye@stu.xjtu.edu.cn (Y.C.); meilichian@aliyun.com (Z.L.); yidasasa047717@126.com (S.Y.); eileen8222@163.com (H.Y.)

**Keywords:** cardiovascular health, stroke, risk factors, epidemiology, prevention

## Abstract

*Background*: In 2010, the American Heart Association developed a new definition of ideal cardiovascular health (CVH) based on seven cardiovascular health metrics. This study aimed to investigate the relationship between modified ideal CVH metrics and the risk of stroke in the rural population of Northeast China. *Methods*: We included 11,417 adults from the rural population in Northeast China and collected all the information, including the baseline characteristics, history of stroke, and the seven ideal CVH metrics. *Results*: Our results showed that the presence of stroke was associated with high body mass index (BMI), poor diet score (salt intake), high total cholesterol (TC), high blood pressure (BP), and high fasting plasma glucose (FPG). The prevalence of stroke increased as the number of ideal CVH metrics decreased, and peaked to 13.1% among those with only one ideal CVH metric. Participants with only one ideal CVH had a 4.40-fold increased susceptibility of stroke than those with all seven ideal health metrics. *Conclusion*: This study revealed that people with a better CVH status had a lower prevalence of stroke and the susceptibility of stroke increased with the decreasing of the number of ideal CVH metrics.

## 1. Introduction

Cardiovascular disease (CVD) has become the leading cause of morbidity and mortality worldwide [1], and stroke is now ranked number 4 in prevalence [2]. Due to the high percentage of functional disability, stroke has been considered as a substantially clinical and socio-economic burden [3]. In China, the number of patients suffering from stroke has increased rapidly because of the longer lifespans, the aging of the population and the modification of lifestyle. In 2007, Liu *et al.*, reported that more than 7 million people in China had suffered from stroke and over 2 million were newly diagnosed per year [4]. Therefore, it is very important to identify the risk factors and to take steps to control stroke in China [5].

In 2010, American Heart Association (AHA) proposed the concept of “ideal cardiovascular health” (CVH), and set national goal for CVH promotion and disease reduction through 2020 and beyond [6]. According to the recommendations, the concept of CVH was based on seven metrics: smoking status, body mass index (BMI), healthy diet score, physical activity level, blood pressure (BP), blood glucose, and total cholesterol (TC). CVH was further classified into three groups; ideal (*i.e.*, people with all seven health metrics at ideal levels), intermediate (having at least one health metric at the intermediate level, but no poor health metrics), and poor (at least one of seven health metrics at a poor level). In subsequent years, many studies worldwide have shown surprisingly low prevalence of ideal CVH among populations [7,8,9,10]. Dong *et al.*, in the Northern Manhattan Study demonstrated a strong inverse relationship between the number of ideal CVH metrics and the total incidence of stroke across whites, blacks, and Hispanics [11]. Recently, Yang *et al.*, also demonstrated that positive changes in ideal CVH metrics reduce the incidence of stroke in a city population in China [12]. However, the association between ideal CVH status and the incidence of stroke in rural Chinese population is still unclear.

Therefore, we performed a cross-sectional study to examine whether ideal CVH metrics were associated with a lower risk of stroke among the rural population of Northeast China. 

## 2. Methods

### 2.1. Study Population

From January 2012 to August 2013, a representative sample of people aged ≥35 years was selected in rural areas of Liaoning Province, which is located in Northeast China. The study adopted a multi-stage, stratified random cluster-sampling scheme. In the first stage of the study, three counties (Dawa, Zhangwu, and Liaoyang County) were selected randomly from rural areas of Liaoning province. In the second stage, one town was randomly selected from each of these three counties. In the third stage, 26 rural villages from the three towns were randomly selected. Individuals who were pregnant, or diagnosed with malignant tumor or mental disorder were excluded from the study. All eligible permanent residents aged ≥35 years from each village were invited to attend the study. The study was approved by the Ethics Committee of China Medical University (Shenyang, China, ethical approved project identification code: 2011-2-2). All procedures were performed in accordance with the University’s ethical standards. Written consent was obtained from all participants after they had been informed of the objectives and benefits of the study, any medical issues and the confidentiality agreement regarding personal information. If the participants were illiterate, we obtained the necessary written informed consent from their proxies. Only those who had a complete set of data were included in the analysis.

### 2.2. Data Collection and Definitions

A standard questionnaire was obtained by face-to-face interview during a single clinic visit with each participant. The questionnaire collected data including the demographic characteristics, lifestyle risk factors, dietary habits, family income, and education levels. 

Before the survey was performed, volunteers were recruited as investigators from medical graduates of China Medical University. All eligible investigators received organized training that covered the purposes of this study, the administering of the questionnaire, the standard method of measurement, the importance of standardization, and the study procedures. Trainees were then given a thorough test and evaluation and only those who passed the test were allowed to become investigators. Additionally, further instructions and support were available during the process of data collection.

The marital status of the participants was categorized into one of two groups: married or living with a partner; or unmarried, divorced, or widowed. Family income was classified as ≤5000, 5000–20,000, and >20,000 CNY/year. Educational level was categorized as low (no schooling, incomplete primary education, and primary education), middle (three or four years of secondary education), and high (college and university education). The subjects were asked whether or not they were currently smoking or drinking alcohol.

The questionnaire also included questions on their average consumption of several food items (including legumes, vegetables, fruits, fish, poultry, and their salt intake) per week. In defining a healthy diet, we originally used the following five components: (1) with legumes and cereals as the basic food; (2) ≥500 g fruits and vegetables/day; (3) <100 g red meat/day; 4 = regular (*i.e.*, in most weeks) intake of soybean products and/or unprocessed fish; and 5 = preference for non-salty food, in accordance with the current “Dietary Guidelines for Chinese Residents” developed by the Chinese Nutrition Society and proclaimed by the Ministry of Health [13]. In the processing of the data, we found that the vast majority of the subjects’ consumption fell within the standard of the Guidelines, with the exception of salt intake. In the end, however, we decided to use salt intake instead of dietary data as the metric, and it was classified into three categories: low, medium, or high salt intake, defined as ≤6 g/day, 6–10 g/day, or >10 g/day, respectively.

It should be noted that, after taking high-intensity agricultural activities into consideration, we classified all study participants as being at the “ideal” physical activity level. In rural areas, men and women, whether they are old or young, typically engage in agricultural activities, from sowing in spring, to harvest in autumn and gathering firewood in winter. The intensity of activity exceeds the AHA criterion that defines ideal physical activity as being 150 min/week of moderate intensity physical activity or 75 min/week of vigorous intensity physical activity or a combination of these.

Weight and height were measured to the nearest 0.1 kg and 0.1 cm, respectively, with the participants wearing lightweight clothing and without shoes. Waist circumference (WC) was measured at the umbilicus using a non-elastic tape (to the nearest 0.1 cm), with the participants standing, and at the end of normal expiration. Body mass index (BMI) was calculated as weight in kilograms divided by the square of the height in meters. BMIs were categorized into three groups according to the World Health Organization (WHO) criteria; as normal (BMI < 25 kg/m^2^), overweight (BMI ≥ 25 kg/m^2^, <30 kg/m^2^) and obese (BMI ≥ 30 kg/m^2^) [14].

Blood pressure was measured three times at 2 min intervals after at least 5 min of rest using a standardized automatic electronic sphygmomanometer (HEM-907; Omron, Dalian, China). The participants were advised to avoid consuming caffeinated beverages and avoid exercise for at least 30 min before the measurement. During the measurement, the participants were seated with the arm supported at the level of the heart. The mean of three blood pressure (BP) measurements was calculated and used in all analyses. In line with the Seventh Report of the Joint National Committee on Prevention, Detection, and Evaluation of High Blood Pressure (the JNC-7 report) [15], hypertension was defined as SBP ≥ 140 mm Hg and/or DBP ≥ 90 mm Hg and/or the use of antihypertensive medications. 

Fasting blood samples were collected in the morning after at least 12 h of fasting for all participants. Blood samples were collected from an antecubital vein into vacutainer tubes containing EDTA. Fasting plasma glucose (FPG), total cholesterol (TC), and other routine blood biochemical indexes were analyzed enzymatically on an autoanalyzer. All laboratory equipment was calibrated and blinded duplicate samples were used.

Dyslipidemia was defined according to the National Cholesterol Education Program-Third Adult Treatment Panel (ATP III) criteria [16]. High TC was defined as TC ≥ 6.21 mmol/L (240 mg/dL). Low HDL-C was defined as HDL-C < 1.03 mmol/L (40 mg/dL). High LDL-C was defined as LDL-C ≥ 4.16 mmol/L (160 mg/dL). High TG was defined as ≥2.26 mmol/L (200 mg/dL). Dyslipidemia was defined as having at least one of the values of high TC, high LDL-C, low HDL-C, and high TG. 

Diabetes mellitus was diagnosed according to the WHO criteria [17] of FPG ≥ 7 mmol/L (126 mg/dL) and/or being on treatment for diabetes.

#### 2.2.1. Modified AHA’s Criteria

In accordance with the definition published previously [6], ideal health is equal to all seven health metrics at ideal levels; intermediate health is at least one health metric at the intermediate level, but no poor health metrics; and poor health is at least one of seven health metrics at poor level. Health behaviors, including smoking, BMI, physical activity, and diet score, were all categorized as ideal, intermediate, or poor. Smoking was classified as ideal (never a smoker or quitting >12 months before), intermediate (quitting ≤12 months before), or poor (current smoker). BMI was classified as ideal (<25 kg/m^2^), intermediate (25–29.9 kg/m^2^), or poor (≥30 kg/m^2^). Healthy diet was classified according to salt intake and classified as ideal (<6 g/day), intermediate (6–10 g/day), or poor (≥10 g/day). Physical activity was classified as ideal for all participants, for reasons previously given. Health factors included smoking, TC, BP, and FBG. TC was classified as ideal (<200 mg/dL, untreated), intermediate (200–239 mg/dL or drug treated to goal), or poor (≥240 mg/dL). BP was classified as ideal (SBP < 120 mmHg and DBP < 80 mmHg, untreated), intermediate (SBP 120–139 mmHg or DBP 80–89 mmHg, or treated to goal), and poor（SBP ≥ 140 mmHg or DBP ≥ 90 mmHg). FPG was classified as ideal (<100 mg/dL, untreated), intermediate (100–125 mg/dL or drug treated to goal), or poor (≥126 mg/dL). 

#### 2.2.2. Stroke

Incidents of self-reported or family-reported stroke were obtained from a questionnaire and all participants who reported an incident of stroke were asked to review their medical records, including reports of brain imaging, such as CT or MRI scanning.

### 2.3. Statistical Analysis

Descriptive statistics were reported as mean values and standard deviations and categorical variables were reported as numbers and percentages. Differences among categories were evaluated using the non-parametric test or the χ^2^-test as appropriate. Multivariable logistic regression analyses were used to identify stroke in relation to cardiovascular health (CVH) metrics with odds ratios (ORs) and corresponding 95% confidence intervals (CIs) calculated. All the statistical analyses were performed using SPSS version 17.0 software, and *p* values less than 0.05 were considered to be statistically significant.

## 3. Results

A total of 11,417 subjects (5271 males and 6146 females) aged ≥35 years participated in the study. Overall, there were 1011 participants with a history of stroke, accounting for 8.9% of our study population. Table 1 shows the demographic and clinical characteristics of the population. Participants suffering from stroke were older than the general population (60 (10) *vs.* 53 (10), *p* < 0.001). Overall, the education level and family income were lower in stroke group than that in the non-stroke group. Participants with stroke had lower smoking and drinking rates and took more salt in their daily diet. In addition, BMI and WC were greater in the stroke group than that in the non-stroke group. Furthermore, SBP, DBP, TC, TG, LDL-C, as well as FPG, were significantly higher in the stroke group compared with the non-stroke group, while the HDL-C values showed the opposite trend.

**Table 1:** Baseline characteristics of study population.

Variables	Total (*n* = 11,417)	Non-Stroke (*n* = 10,406)	Stroke (*n* = 1011)	*p*-Value
Age (year)	53 (11)	53 (10)	60 (10)	<0.001
Male (%)	46.17	45.90	48.80	<0.001
Spouse (live, %)	91.62	91.98	87.83	<0.001
Education (%)				<0.001
Primary school or below	49.77	48.58	62.02	
Middle school	40.83	41.72	31.65	
High school or above	9.41	9.70	6.33	
Family income (CNY/year, %)				<0.001
≤5000	12.41	11.55	21.26	
5000–20,000	54.48	54.36	55.69	
>20,000	33.11	34.09	23.05	
Salt intake	6.38 (3.60)	6.36 (3.58)	6.62 (3.73)	<0.001
Current smoking status (%)	35.39	35.50	34.52	<0.001
Current drinking status (%)	22.54	23.44	13.25	<0.001
SBP (mmHg)	141.76 (23.48)	140.49 (22.71)	154.76 (27.04)	<0.001
DBP (mmHg)	82.05 (11.78)	81.64 (11.62)	86.30 (12.63)	<0.001
BMI (kg/m^2^)	24.80 (3.66)	24.75 (3.66)	25.36 (3.70)	<0.001
WC (cm)	82.43 (9.83)	82.18 (9.79)	85.00 (9.89)	<0.001
TC (mmol/L)	5.23 (1.09)	5.21 (1.08)	5.45 (1.12)	<0.001
TG (mmol/L)	1.63 (1.50)	1.61 (1.48)	1.92 (1.64)	<0.001
LDL-C (mmol/L)	2.92 (0.82)	2.91 (0.82)	3.09 (0.85)	<0.001
HDL-C (mmol/L)	1.41 (0.38)	1.41 (0.38)	1.34 (0.35)	<0.001
FPG (mmol/L)	5.91 (1.64)	5.86 (1.58)	6.36 (2.09)	<0.001

Data are expressed as the mean (SD) or as *n* (%). Abbreviations: CNY, China Yuan (1 CNY = 0.161 USD); SBP, systolic blood pressure; DBP, diastolic blood pressure; BMI, body mass index; WC, waist circumference; TC, total cholesterol; TG, triglyceride; LDL-C, low-density lipoprotein cholesterol; HDL-C, high-density lipoprotein cholesterol; FPG, fasting plasma glucose.

The distribution of CVH metrics in our study population is shown in Table 2. Of all the participants, 63.8% had never smoked or had quit smoking for more than 12 months, 55% had a BMI <25 kg/m^2^, 56.4% took less than 6g salt in their daily diet, 52.2% had untreated total cholesterol <200 mg/dL (treated to goal), 15.5% had untreated blood pressure <120/<80 mm Hg (treated to goal), and 50.5% had untreated fasting serum glucose <100 mg/dL (treated to goal). Overall, compared to the stroke group, a much higher proportion of participants in the non-stroke group met ideal levels for each CVH metric, except for smoking status.

**Table 2:** Distribution of cardiovascular health metrics.

Metric	Definition	Total, %	Non-Stroke, %	Stroke	*p*-Value
		(*n* = 11,417)	(*n* = 10,406)	(*n* = 1011)	
**Current smoking**					<0.001
Ideal	Never or quit >12 months	63.8	63.7	64.7	
Intermediate	Former ≤12 months	0.8	0.8	0.8	
Poor	Current	35.4	35.5	34.5	
**Body mass index**					<0.001
Ideal	<25 kg/m^2^	55.0	55.7	47.5	
Intermediate	25−29.99 kg/m^2^	37.3	36.8	41.9	
Poor	≥30 kg/m^2^	7.7	7.5	10.6	
**Salt intake**					<0.001
Ideal	<6 g	56.4	56.8	51.6	
Intermediate	6−10 g	32.6	32.3	36.4	
Poor	≥10 g	11.0	10.9	12.0	
**Total cholesterol**					<0.001
Ideal	<200 mg/dl ^a^	52.2	53.0	44.0	
Intermediate	200−239 mg/dl ^b^	30.8	30.5	33.3	
Poor	≥240 mg/dl	17.0	16.5	22.7	
**Blood pressure**					<0.001
Ideal	<120/80 mm Hg ^a^	15.5	16.4	6.4	
Intermediate	SBP 120−139 or DBP 80−89 mm Hg ^b^	36.5	37.7	23.4	
Poor	SBP ≥ 140 or DBP ≥ 90 mm Hg	48.0	45.8	70.1	
**Fasting plasma glucose**					<0.001
Ideal	<100 mg/dl ^a^	50.5	51.5	40.3	
Intermediate	100–125 mg/dl ^b^	39.9	39.7	41.8	
Poor	≥126 mg/dl	9.6	8.8	17.9	

^a^ or without medication; ^b^ or treated to goal.

After adjustment for age, sex, spouse, education, family income, and drinking, the presence of stroke was associated with poor BMI (OR: 1.73, 95% CI: 1.44–2.06); poor diet score (salt intake) (OR: 1.23, 95% CI：1.04–1.44); poor TC (OR: 1.55, 95% CI: 1.36–1.78); poor BP (OR: 1.68, 95% CI: 1.43–1.98); and poor FPG (OR: 1.82, 95% CI: 1.55–2.15), as shown in Table 3. However, the presence of stroke was adversely associated with current smoking (OR: 0.68, 95% CI: 0.61–0.76).

Prevalence of stroke showed a graded correlation to the number of ideal CVH metrics after adjustment for age, spouse, education, family income, and drinking status. As shown in Table 4, only five individuals suffered from stroke among those who met all seven ideal health metrics. Stroke prevalence was very low among participants with 5–7 ideal CVH metrics (5.9%, 4.9%, and 1.6%, respectively). The prevalence of stroke increased as the number of ideal CVH metrics decreased, and peaked to 13.1% among those with only one ideal CVH metric. After adjustment for age, spouse, education, family income, and drinking status, the susceptibility of stroke increased with the decreasing of the numbers of ideal CVH metrics. Participants with three, two, and one ideal CVH metrics had 3.60- (95% CI: 1.46–8.85), 4.03- (95% CI: 1.62–10.00) and 4.44-fold (95% CI: 1.66–11.79) susceptibility of stroke, compared with participants who met all seen ideal health metrics.

**Table 3:** Odds ratio of stroke based on the cardiovascular health metrics.

Metrics	Odds Ratio	95% CI
Current smoking		
Ideal	1.00	Reference
Intermediate	1.08	0.64–1.84
Poor	0.68	0.61–0.76
Body mass index		
Ideal	1.00	Reference
Intermediate	1.26	1.13–1.41
Poor	1.73	1.44–2.06
Salt intake		
Ideal	1.00	Reference
Intermediate	1.11	0.99–1.24
Poor	1.23	1.04–1.44
Total cholesterol		
Ideal	1.00	Reference
Intermediate	1.17	1.04–1.31
Poor	1.55	1.36–1.78
Blood pressure		
Ideal	1.00	Reference
Intermediate	1.13	0.95–1.34
Poor	1.68	1.43–1.98
Fasting plasma glucose		
Ideal	1.00	Reference
Intermediate	1.29	1.15–1.44
Poor	1.82	1.55–2.15

According to the categories of ideal CVH defined by the AHA, the prevalence of stroke was 1.3% among participants with ideal CVH, 4.2% among those with intermediate CVH and 10.2% among those with poor CVH. Individuals with poor CVH thus had a 4.40-fold (95% CI: 1.62–11.91) higher odds of stroke, compared with those with ideal CVH, see Table 5.

## 4. Discussion

This cross-sectional study found that people in the rural population of Northeast China with a better status of CVH had a lower prevalence of stroke. The prevalence of stroke correspondingly increased with the decreasing of the numbers of ideal CVH metrics. Individuals with poor CVH had a 4.40-fold (95% CI: 1.62–11.91) higher odds of stroke compared with those with ideal CVH.

The results were consistent with the previous studies concerning the association between CVH metrics and stroke [11,12,18,19]. According to the AHA definition, CVH metrics consist of health behaviors (smoking status, BMI, healthy diet score, and physical activity level) and health factors (BP, TC, and FGP). Previous studies have shown that the combined effect of some healthy lifestyle factors was associated with stroke [18,19]. In these studies, a healthy lifestyle pattern was similarly defined as nonsmoking, BMI < 25 kg/m^2^, ≥30 min/d of moderate activity, a favorable overall diet quality, and moderate alcohol consumption. More health behaviors were substantially associated with lower risks for stroke in both sexes [11,18,19]. Similar graded relationships were also observed in our study. More recently, Yang *et al.*, reported a similar result in a city population in China indicating that positive changes in ideal CVH metrics could reduce the incidence of stroke during a 4.89-year follow-up period [12], which was in consistent with our findings.

**Table 4:** Prevalence and odds ratios of stroke according to number of ideal CVH metrics ^a^.

Number of Ideal CVH Metrics	Number of Stroke	Total Number (%) in Category	% Stroke in Category	Odds Ratio (95% CI)
7	5	307 (2.7)	1.6	Reference
6	54	1099 (9.6)	4.9	2.24 (0.88, 5.69)
5	138	2331 (20.4)	5.9	2.26 (0.91, 5.61)
4	312	3305 (28.9)	9.4	3.27 (1.33, 8.034)
3	316	2851 (25.0)	11.1	3.60 (1.46, 8.85)
2	156	1295 (11.3)	12.0	4.03 (1.62, 10.00)
1	30	229 (2.0)	13.1	4.44 (1.66,11.79)

^a^ adjusted for age, marital status, education, family income and drinking status.

**Table 5:** Prevalence and odds ratios of stroke according to CVH categories ^a^.

Categories of CVH	Number With Stroke	Total Number (%) in Category	% Stroke in Category	Odds Ratio (95% CI)
Ideal ^b^	4	303 (2.7)	1.3	Reference
Intermediate ^c^	103	2453 (21.5)	4.2	2.41 (0.88, 6.62)
Poor ^d^	904	8661 (75.9)	10.2	4.40 (1.62, 11.91)

^a^ adjusted for age, marital status, education, family income, and drinking status; ^b^ all seven health metrics at ideal levels; ^c^ at least one health metric at intermediate level, but no poor health metrics; ^d^ at least 1 of seven health metrics at poor level.

In the processing of statistical data, we found that the vast majority of the participants could meet the AHA’s recommended standard healthy diet score, except for salt intake. High salt intake was an important risk factor for hypertension [20,21], which was closely related to the presence of stroke [22,23]. Therefore, in line with other Chinese studies [24,25], we took salt intake as a replacement for the diet score. Our results confirmed that participants with poor level of salt intake had a 1.23-fold higher odds of stroke.

However, inconsistent with other studies in which smoking was a doubtless risk factor for stroke [6,18,19], smoking showed a protective effect in our study. We speculated that perhaps participants who suffered from stroke would stop smoking on their own or be persuaded by family members, which would explain why current smokers were fewer in the stroke group than that in the non-stroke group. However, this is the shortcoming of cross-sectional study that could not identify the sequential relationship of smoking and stroke. 

Furthermore, the percentage of current smokers was 35.4% in our study, similar to two other studies in China (prevalence 28.2% and 24.6%, respectively) [26,27], and higher than that in Western countries ranging from 10.0% to 17.3% [28,29,30]. As discussed above, smoking was, without doubt, a risk factor for stroke [6,18,19], and the tobacco epidemic in China was more severe than in other developed countries. Therefore, controlling smoking was much more beneficial for the decreased presence of stroke. 

Health factors consisted of BP, TC, and FGP, which were further classified into three levels as ideal, intermediate and poor. Previous studies have indicated that hypertension [4,20,21] (poor level of blood pressure), hyperlipidemia [31,32] (poor level of TC), and diabetes mellitus [33,34] (poor level of FGP) were all independent risk factors for stroke. Our results were consistent with the above studies. Our study showed that 48.0% of the rural population had a poor level of blood pressure and it was much higher in the stroke group (up to 70.1%) than the non-stroke group, which was consistent with other studies from China. One cross-sectional study based on the general population aged 18–79 found that the prevalence of hypertension in Beijing was 35.5% and increased with age [29]. Li *et al.*, in another cross-sectional study indicated that 43.8% of the rural population had hypertension in Shandong Province, China [35]. The prevalence of hypertension in China was much higher than that in Western countries in which it ranged from 18% to 28.3% [30,36,37].

We also found that these risk factors had a dose-response relationship for the presence of stroke and the odds ratio of stroke correspondingly increased with decreasing numbers of ideal CVH metrics. In a four-year-follow-up study from China, Zhang *et al.*, found that participants who met more than six ideal CVH metrics were 76% less likely to develop stroke compared with those who had poor CVH [38]. Dong *et al.*, in the Northern Manhattan Study demonstrated a strong inverse relationship between the number of ideal CVH metrics and the total incidence of stroke across whites, blacks, and Hispanics [11]. Consistently, in the ARIC study, Hozawa *et al*., [39] estimated that 70% of coronary heart disease (CHD) and stroke events might be eliminated if participants avoided the above factors.

Overall, health promotion measures, including a low salt diet, weight control, smoking cessation, antihypertensive, antidiabetic and lipid-lowering therapy, would increase the prevalence of ideal CVH and therefore lead to a lower risk for stroke [11].

## 5. Limitations

Our study has some important limitations. First, as a cross-sectional study, it was difficult to establish a causal relationship between the ideal CVH metrics and stroke. Second, although the present study achieved a high respond rate and could be representative of the local population, the selection of each individual was not completely randomized. The selection bias could compromise the results to some extent. Third, measurement of CVH behaviors—physical activity and diet score—were modified slightly in our study and might not be as valid and reproducible as in others.

## 6. Conclusions

This study revealed that people with a better CVH status had a lower prevalence of stroke and the prevalence of stroke increased with the decreasing of the number of ideal CVH metrics. 

## Abbreviations

The following abbreviations are used in this manuscript:

CVH: Ideal Cardiovascular Health
CVD: Cardiovascular Disease
AHA: American Heart Association
BP: Blood Pressure
TC: Total Cholesterol
FPG: Fasting Plasma Glucose
WC: Waist Circumference
BMI: Body Mass Index
WHO: World Health Organization
CHD: Coronary Heart Disease
